# A structured comparison and reflection on international position statements and professional guidance for the management of periviable infants

**DOI:** 10.3389/fped.2025.1553033

**Published:** 2025-05-15

**Authors:** J. Peterson, G. Southwood, D. M. Smith, E. D. Johnstone, A. Mahaveer

**Affiliations:** ^1^ Faculty of Biology, Medicine and Health, The University of Manchester, Manchester, United Kingdom; ^2^ Neonatal Intensive Care Unit, St Mary’s Maternity Hospital, Manchester University NHS Foundation Trust, Manchester, United Kingdom; ^3^ Maternity Services, St Mary's Hospital, Manchester University NHS Foundation Trust, Manchester, United Kingdom

**Keywords:** neonates, extreme preterm, perinatal, birth, palliative care

## Abstract

**Background:**

Survival rates and clinical approach to periviable infants are rapidly evolving at certain centres, but there remains variation in definition, approach and management of these infants worldwide. This review aims to narratively review and discuss professional guidelines, position statements and frameworks for management of periviable infants (22 + 0–25 + 0 weeks gestation) born in countries with the highest relative spending on healthcare.

**Methods:**

Eligible countries were determined using the Organisation for Economic Co-operation and Development database. The top 10 countries with highest spend on healthcare as a proportion of their gross domestic profit were selected. A comprehensive search of relevant databases and search engines (MEDLINE, Embase, CINAHL, PsycINFO, Google Scholar) was performed to identify professional guidance documents for each eligible country. The primary outcome was the delivery room management recommendation (survival-focused or end of life care). The secondary outcomes were survival rates, disability rates and whether shared decision-making with parents was recommended.

**Results:**

There was variation in definition of periviable and approach to management across the 10 professional guidelines. There was a four-week difference across countries for where the limits of viability lie (22 + 0–25 + 6 weeks). At 22-weeks, eight guidelines recommended comfort care and only one country recommending active care as the default management position at birth. By 24-weeks gestation, no country recommended comfort care as the standard approach at birth.

**Discussion:**

Despite the included countries having the highest spend on healthcare as a proportion of their GDP, there is marked international variation in recommended practice in relation to the definition of and management for periviable infants. The majority of included guidelines recommended a shared decision-making approach between professionals and parents facing periviable birth, however, there were scant details about how this should be actualized and only two guidelines included decision-making aids for use with parents. The pre-birth discussion between perinatal professionals and parents facing periviable labour is complex and challenging for all involved. Further research is required to explore how best to facilitate parental understanding and involvement in these discussions to ensure parents are empowered to make the most appropriate decisions for their baby and their family.

## 1 Introduction

There is increasing focus and research on the ethically complex area of management of infants born in the periviable period. This period refers to the timepoint in the pregnancy where it may be possible, albeit improbable, that the infant could survive outside the womb with provision of intensive medical care. Where this timepoint lies depends on the combination of the medical, technological and societal context the mother-infant dyad exists within. The gestational age range at which the pregnancy is considered periviable has therefore, varied throughout history and across geographical location and associated differing healthcare systems. For example, in the United Kingdom sequential national frameworks for management of the extremely preterm infant have moved from consideration of intensive care from 24 + 0 weeks to the revised framework, released in 2019, outlining that active, survival-focused treatment could be considered from 22 + 0 weeks (1, 2).

Due to the risks of death and, for survivors, the risks of long-term physiological and neurodevelopmental impairments, the decision to provide intensive care to periviable infants is not straightforward and requires consideration of numerous practical and ethical elements of care. Approaches and outcomes described in the literature and presented at academic meetings vary considerably between centres and across countries (3). Long term data regarding developmental outcomes of survivors is limited and often evaluated using endpoints or scoring systems which lend themselves to quantitative analysis but may not be conducive to functionally meaningful interpretation by parents and practising professionals (4). More information is needed about functional and social outcomes over the course of childhood (5). Additionally, data regarding the outcomes of extreme preterm infants who have survived into adulthood is inherently problematic due to the required time lag which then reflects historical neonatal practice (6). Provision of intensive care to periviable infants requires prolonged hospital stays, access to specialist equipment, medications and multidisciplinary expertise. Consequently, care of the preterm infant is extremely expensive in the neonatal period with costs increasing the more premature the infant is (7, 8). Given the cost implications, provision of care may be affected by the country and healthcare system the infant is born into. This review aims to summarise and present the approaches and recommended options for management at birth for periviable infants across countries with highest relative spending on healthcare.

## 2 Methods

The study protocol was developed in accordance with the international Appraisal of Guidelines, Research and Evaluation (AGREE) process (9). The study protocol was pre-registered with PROSPERO (CRD42022300099) (10).

### 2.1 Search strategy

Countries for inclusion in this review were selected using the Organisation for Economic Co-operation and Development (OECD) database to identify the top ten countries with highest spend on healthcare as a percentage of their gross domestic profit (GDP) (11).

**Figure 1:**
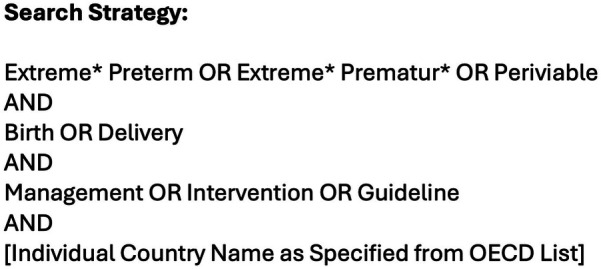
Search strategy.

The PICOC (Population, Intervention, Comparison, Outcome, Context) framework was used as a conceptualising framework to formulate the review question and search process. The PICOC elements were outlined: Population = Periviable Infants; Intervention = Management at Delivery (survival-focused or comfort care at delivery); Comparison = N/A; Outcome = Recommendation as stated in the guideline; Context = High-income countries. Boolean operators were utilised to combine keywords and blocks. The search strategy (Figure 1) was developed by JP and GS with support from a third reviewer DMS. The search strategy and inclusion/exclusion criteria were agreed prior to conducting the search. The MEDLINE® (OVID), Embase™ (OVID), CINAHL (EBSCO), PubMed and Google/Google Scholar databases were searched using the pre-agreed search strategy (Figure 1).

### 2.2 Inclusion criteria

• Professional Guideline or Framework or Commentary outlining the country's approach
• Content focused on approach to management of infants born at 22 + 0–24 + 6 weeks
• Countries listed in the OECD top ten in 2022 for highest spending on healthcare as %GDP
• Articles written in English or language available in Google Translate to allow translation

### 2.3 Exclusion criteria

• Any other article type or secondary analysis of other studies (summaries, systematic reviews, meta-analyses)
• Exclusively focused on management of infants < (and including) 21 + 6 weeks or >  (and including) 25 + 0 weeks
• Related to country not on the 2022 OECD listing for top ten for highest spending on healthcare as %GDP
• Written in language unavailable through Google Translate

### 2.4 Study selection and critical appraisal

The OECD online database was searched for list of countries with highest spend (% GDP) on healthcare. At the time of the search (April 2024), the most recent OECD data was from 2022; therefore, this was the list of countries that was used for this review.

The search strategy (Figure 1) was then utilised substituting in each of the top ten identified countries from the OECD listing. The results were then manually screened by JP and GS to identify either the professional guideline from the respective county's national neonatal organisation (for example, the British Association of Perinatal Medicine) or for the most comprehensive consensus statement from neonatal professionals outlining their national approach to periviable birth. Both reviewers (JP and GS) had to be in agreement for the selected guideline to be included in the review (Kappa 1.0). All ten identified guidelines were screened against the inclusion and exclusion criteria by JP and GS. There was a third author (DMS) available in the event of any disagreement; this was not required. The most recent version of the guideline from each country was included in the review. For any publications not available in English, Google Translate was used to translate these articles in English for analysis. The search was performed on 23rd April 2024.

### 2.5 The analysis process

The primary outcome was the pre-delivery recommendation of intensive or comfort care at birth. The secondary outcomes were inclusion of survival rates, disability rates and whether shared decision-making with parents was recommended.

Data from included guidelines (country of publication, year of publication, primary outcome, secondary outcome, denominator used in the guideline, conclusions and limitations) was extracted and summarised into a data extraction matrix for analysis and comparison of key points and recommendations from across the guidelines. All included guidelines were appraised using the AGREE II evaluation tool by JP and GS (12). The AGREE II scores were performed independently by JP and GS and the scaled domain scores calculated using the AGREE II tool.

### 2.6 Ethical consideration

This study is a review of previously published guidelines and therefore, did not require formal ethics approval. All included guidelines were conducive with the Declaration of Helsinki.

## 3 Results

The search identified a combination of professional guidelines, frameworks and consensus statements regarding the management of extremely preterm infants considered to be at the limits of viability (1, 13–20). Where that limit lies varies by country (Table 1). Eight of the ten guidelines included data about survival within the guideline and seven of the ten included data regarding disability rates. Each included guideline was assessed for quality using the AGREE II tool (Table 2). Quality of the guidelines was variable, reflective of their heterogenous approach, scope and depth of included information.

**Table 1:** Overview of the included guidelines and recommendations.

Country	Name of guideline	Release/latest review date	Gestational age			Terminology used in relation to comfort care option (exemplar quotes, if stated)
			22 + 0–22 + 6	23 + 0–23 + 6	24 + 0–24 + 6	
United States	Antenatal Counselling Regarding Resuscitation and Intensive Care Before 25 Weeks of Gestation	2015	Individual, holistic assessment	Individual, holistic assessment	Individual, holistic assessment	If a decision is made not to resuscitate, providing comfort care, encouraging family bonding, and palliative care support are appropriate.
Germany	Premature babies at the limit of viability	2020	If weight < 400 g: Comfort Care	If weight < 400 g: Intensive care if parents decision	If weight < 400 g: Individual, holistic assessment	The main prerequisite for this is that the child does not suffer as a result. There is an obligation to ensure adequate medical care for both primary life-prolonging and primary palliative treatment goals.
			If weight > 400 g: Intensive care if parents decision	If weight > 400 g: Individual, holistic assessment	If weight > 400 g: Intensive care (unless other severe risk factors)	
France	Very premature births: Dilemmas and management. Second part: Ethical aspects and recommendations	2010	Comfort Care	Comfort Care	Individual, holistic assessment	Withholding intensive care at birth for babies born below or within the gray zone does not mean withholding care but rather providing palliative care to prevent pain and suffering during the time period preceding death.
Japan	Neonatal Intensive Care Manual for the infants born less than 28 weeks of gestation	2019	Intensive care	Intensive care	Intensive care	Note: No use of the terms “palliative”, “comfort” or “end of life”. Some references to “death” in the context of death being a potential clinical outcome.
Austria	Medical care for premature babies at the limit of viability	2016	Comfort Care	Individual, holistic assessment	Intensive care	Section 4.1: Palliative care: If a decision is made to change the therapeutic goal to “palliative care” because the continuation of life-sustaining intensive medical treatments is no longer indicated under the given circumstances, everything must be done to alleviate pain, shortness of breath and other suffering as best as possible… If parents wish, they should be able to say goodbye and grieve easier by having close contact with the dying child for as long as possible. If the parents want, the child should be allowed to die in the arms of its mother or father.
United Kingdom	Perinatal Management of Extreme Preterm Birth before 27 weeks of gestation	2019	Comfort Care (consideration of survival-focused care if favourable factors/parental preference)	Comfort Care (consideration of survival-focused care if favourable factors/parental preference)	Intensive care (unless unfavourable risk factors and parental agreement for Comfort Care)	…the terms “active care (survival focused)” [have been used] to refer to obstetric and neonatal management that has the aim of sustaining life for the baby, and “palliative care (comfort focused)” to refer to obstetric and neonatal management when the aim is not to attempt to sustain the life of the fetus/baby, but to focus on the baby's comfort.
Switzerland	Perinatal care at the limit of viability between 22 and 26 completed weeks of gestation in Switzerland	2011	Comfort Care	Individual, holistic assessment	Intensive care	The members of the interdisciplinary working group suggest that the care of preterm infants with a gestational age between 22 0/7 and 23 6/7 weeks should generally be limited to palliative care.
New Zealand	New Zealand Consensus Statement on the care of mother and baby(ies) at periviable gestations	2019	Comfort Care	Individual, holistic assessment	Individual, holistic assessment	At 23^+^⁰ to 24^+^⁶ weeks, options to be considered should include active treatment and supported comfort/palliative care.
Canada	Counselling and management for anticipated extremely preterm birth	2023	Comfort Care	Individual, holistic assessment	Individual, holistic assessment	Depending on an infant's prognosis, healthcare professionals usually present parents with one or two broad management options during a prenatal consultation: early intensive care (with ongoing re-evaluation) and/or palliative care.
Belgium	Recommendation regarding perinatal concerns regarding viability in Flanders	2014	Comfort Care	Comfort Care	Individual, holistic assessment	…then speak of palliative or comfort care and that means that we do everything possible to ensure that the baby is calm and pain-free during this short period of time in the world without aggressive life-saving measures.

**Table 2:** AGREE II scoring tool.

Country	Scope & purpose	Stakeholder involvement	Rigor of development	Clarity of presentation	Applicability	Editorial independence	Overall guideline assessment	Overall guideline assessment (recommendation)
USA	83%	56%	36%	44%	33%	71%	4	No
Canada	89%	83%	72%	64%	31%	21%	5	Yes
Germany	83%	78%	77%	72%	56%	92%	6	Yes
France	67%	64%	21%	28%	17%	92%	3	No
UK	89%	78%	50%	67%	60%	38%	6	Yes
Switzerland	83%	58%	42%	78%	54%	92%	5	Yes
Austria	78%	44%	29%	44%	29%	100%	3	No
New Zealand	83%	72%	49%	69%	50%	58%	5	Yes
Japan	81%	67%	53%	75%	42%	50%	3	No
Belgium	72%	58%	39%	56%	29%	33%	3	No

	Excellent (>80%)	Good (60%–79%)	Average (40%–59%)	Fair (20%–39%)	Poor (<20%)			

Values are the sum of individual scores of criteria within each of the six independent domains scaled as a percentage of the highest possible score. The overall guideline assessment score is rated from 1 (lowest possible quality) to 7 (highest possible quality).

None of the guidelines reviewed recommended resuscitation at less than 22 weeks gestation. Most guidelines acknowledged the diagnostic uncertainty in antenatal assessment of gestational age and weight, and that gestational age alone does not translate to viability, making decision making even more complex. The included guidelines were varied in their date of publication (or last review) from 2011 [Switzerland (18)] through to 2023 [Canada (22)]. Nine of the ten guidelines advocated for a shared decision-making approach between perinatal professionals and parents.

When reviewing the guidelines, the primary outcome was assessment of whether comfort care or intensive care was the recommended management option at birth. Regarding initial management at birth, several guidelines recommendation a holistic assessment of the infant's own risk factors, specific clinical circumstances and parental stance, rather than a predetermined recommendation for either comfort or intensive care. In these cases, this holistic approach has been recorded in Table 1 as a third category, “individualised, holistic assessment”. For guidelines recommending approaches of comfort or intensive care, this does not mean individual risk factors and circumstances are not relevant; indeed, to provide high quality care these factors certainly should be considered. However, the three recommendation categories stated in Table 1 (intensive case, holistic assessment or comfort care) reflect the clarity with which the recommendation is stated within the specific guideline. Where guidelines did not state an approach for an earlier gestation age range, for example, the absence of recommendation for 22 week infants in the New Zealand guideline (19), then the assumption in this review has been that a comfort care approach would be undertaken.

The following sections detail the main findings of the review which were i. the differences in approach to management of the periviable infant at birth, ii. variation in definition of the “periviable” period between countries and iii. use of decision-making tools.

### 3.1 Approaches to management at birth

Recommendation for management at birth varied by gestational age and country (Table 3). For infants born at 22 weeks, eight countries recommended comfort care, one country [USA (13)] recommended an individualised assessment to determine management and one country [Japan (16)] had a default position for intensive care provision. At 23 weeks, the approaches changed with six countries advocating for individualised assessment to determine management, three countries continuing to outline comfort care as recommended management and one country [Japan (16)] implementing intensive care. At 24 weeks, no guidelines had comfort care as recommended management at birth. Instead, six advocated for individualised holistic assessment at birth and four stated intensive care provision would be the standard approach to infants at this gestational age, unless there were significant risk factors for an unfavourable outcome in which case parental values and input would be integrated to guide management at delivery.

**Table 3:** Survival and disability rates by gestational age and associated recommendation as presented in each of the included guidelines (S = survival rates; D = disability rates; R = recommendation from the guideline. For recommendations: CC, comfort care; IHA, individual holistic assessment; IC, intensive care).

Country	Gestational age								
	22 + 0–22 + 6			23 + 0–23 + 6			24 + 0–24 + 6		
	S	D	R	S	D	R	S	D	R
United States	N/A	85%–90%	IHA	N/A	N/A	IHA	N/A	N/A	IHA
Germany	17%–32%	42%	CC	17%–32%	42%	IHA	29%–80%	32%	IHA
France	N/A	N/A	CC	N/A	N/A	CC	N/A	N/A	IHA
Japan	60%–80%	Reported separately by neurodevelopmental domain on the Japanese Neonatal Network website	IC	85%	Reported separately by neurodevelopmental domain on the Japanese Neonatal Network website	IC	85%	Reported separately by neurodevelopmental domain on the Japanese Neonatal Network website	IC
Austria	0%	98%–100%	CC	44%	N/A	IHA	69%	N/A	IC
United Kingdom	30%	24%–43%%	CC	40%	16%–33%	CC	60%	11%–24%	IHA
Switzerland	0%	70%–80%	CC	4%	27%–52%	IHA	31%	22%–44%	IC
New Zealand	N/A	N/A	CC	30%	5%–16%	IHA	60%–65%	13%–20%	IHA
Canada	18%	31%	CC	41%	17%	IHA	67%	21%	IHA
Belgium	0%	N/A	CC	0%–14%	N/A	CC	40%–56%	N/A	IHA

### 3.2 The grey zone

The different countries within this review had variable definitions about the gestational age range that corresponded to the grey zone of decision-making, where, due to mortality and morbidity considerations, there would not be an assumption that intensive care should be the default management approach at birth. The Japanese approach is for intensive care provision as standard for infants born at, or after, 22 + 0 weeks (21). For other countries, such as the United Kingdom, there was a professional recommendation for comfort care whilst acknowledging that intensive care could be provide from 22 + 0 weeks following holistic assessment and parental input (1). Conversely, guidelines from other countries, such as New Zealand (19), did not address management of birth between 22 + 0–22 + 6 weeks, instead framing the guideline around a definition of periviablity as being birth from 23 + 0–24 + 6 weeks, and other countries, such as France (15), extending this further by defining the grey zone as between 24 + 0–25 + 6 weeks gestation.

### 3.3 Decision-making tools

Nine of the ten guidelines encourage use of a shared decision-making approach between parents and perinatal professionals. Shared decision making requires integration of professional knowledge and parental values. This can be difficult to actualize due to potentially limited timeframe from the onset of labour to delivery and the complexity of the risk information that parents and professionals need to navigate. For these reasons, there was inclusion of decision-making aids within several of the guidelines. Decision-making aids varied between guidelines in terms of the target audience and included information. Guidelines such as the Canadian and Swiss guidelines (18, 22), included summary tables for professionals outlining key recommendations, rationale and role of parental input into the decision-making process. There were only two guidelines [UK and New Zealand (1, 19)] which included decision-making aids for use with parents. In both cases, these parent-focused decision aids were visual representations of the survival and morbidity rates by gestational age using live births at the relevant gestational age as the denominator.

## 4 Discussion

Periviable birth raises many ethical and practical difficulties for perinatal professionals. These complexities are reflected in the varied approaches to periviable birth management seen in this international review. The following sections will discuss the impacts of interactional variation in practice and use of parent-facing decision-making tools, alongside differences and implications of the terminology used within these guidelines.

### 4.1 Variation in approach

The included guidelines highlight the geographical variation that is present across international approaches to management of infants born extremely premature from high-income countries. In an age of international professional conferences, electronic journals, online meetings and the potential for sharing clinical experience and expertise that this brings, it seems at odds that there is not one shared definition amongst high-income countries of which infants constitute being considered periviable (or born within the grey zone). The limits of what is possible within a given healthcare system are linked, in part, to the economic provision for healthcare, and in this circumstance, spending on perinatal care and services. However, for countries with similar spending on healthcare it is curious that across these countries the gestational age range within in which the grey zone falls spans a four-week period, from 22 + 0 through to 25 + 6 weeks. Whilst gestational age is not the only factor dictating the limits of viability - and there is increasing focus on perinatal professionals making a holistic, rather than gestational age based, assessment – anyone who has provided care to a 22 week infant will recognise how distinctly different they are to a 25 week infant, and this difference is reflected in the varied survival and morbidity outcomes cited across many of the included guidelines. Infants and their families do not exist in isolation and are a part of the culture of the society within which they live and none of us live in a homogenous world (23). There is large variation in culture, environment, social constructs and expectations, perceptions of the self and the family within the social context, family structures and genetics. This variation creates different cultures, practices and innovations. It would be counterproductive to insist that all countries finance and approach healthcare in the same way; rather it is important for populations to match their healthcare provision to their population's priorities and needs. However, this can cause disparity between options and clinical approaches offered by different countries. Lack of consensus across the high-income countries included in this review about where the grey zone lies means, despite being born into economically similar countries and comparably financed healthcare systems, an infant born at 23 weeks in Japan faces starkly different management than an infant born in France. This is not to suggest that all countries should be forced to have the exact same approach to ethically complex areas of care. Rather, we raise this issue to suggest that given advances in technologies and social networks, these variations in approaches are likely to be accessible to parents facing periviable birth and perinatal professionals should be cognisant that worldwide, amongst otherwise economically comparable countries, there is variation in practice and ethical stance.

The variation in the date each guideline was released (or updated) also highlights the issue of whether professional guidelines seek to reflect clinical practice as done or to distill advances in practice and encourage evolution of care based on the latest research. Given the increasing survival rates and reasonable survival without major morbidity rates from centres with a proactive approach to management of infants from 22 + 0 weeks, it is striking that several of the professional guidelines were published nearly a decade ago.

### 4.2 Use of parent-facing decision-making tools

The pre-birth discussion between perinatal professionals and parents facing periviable labour is complex and challenging for all involved. There is a significant amount of information that parents need access to in order to be meaningfully informed and able to participate in decisions for their baby. Decision-making aids aim to provide a useful adjunct to discussions allowing visual methods of presenting and summarising relevant aspects of the data and factors in the decision-making process. However, from the decision-making aids it can be difficult for parents and perinatal professionals to be able to determine what population the presented data represents. There can be clinically significant differences in outcomes when evaluating different datasets for the same cohort of infants with potential for variation in the same outcome measures across local, regional, national and international datasets (24, 25). Whilst interpreting data and outcome statistics is inherently problematic when attempting to make decisions for an individual infant, the outcome data does provide a context, influencing discussions and likelihood decision-making. Local data may not be available as the centre may have such limited numbers of cases that interpretation of the data would be devoid of much meaning. However, outcomes can vary by region and, as this review demonstrates, clinical approaches vary internationally, making it pertinent for parents and perinatal professionals to be able to rapidly determine which data are being presented to them and where this has been derived from. This level of detail is absent from the British Association of Perinatal Medicine (BAPM) infographic page (26) and in the case of the New Zealand decision-making tool, the source data is cited as the Canadian Society position statement, thus presenting outcome data from a population of infants from a different continent, society and healthcare system. Additionally, for morbidity outcomes there are varying follow-up and assessment practices. For the data presented in the BAPM infographic there is no detail about at what age the assessments of “severe disability” were performed. Within the BAPM framework document itself [(1) page 20–21] there are various studies presented related to the assessment of severe disabilities at ages 2.5–10 years old with the cited research having been performed between 1995 and 2006. However, this section of the framework is not in the parent information section, and the infographic which is aimed at being used by parents, does not provide any context or descriptors of what constitutes severe disability'; there is a sentence at the bottom of the infographic page which states (in pale gray) “*Up to a quarter of children without severe disability may nonetheless have milder forms of disability such as learning difficulty, mild cerebal palsy or behavioural problems*.” (26). The New Zealand decision-making aid makes it clear within the aid itself that the developmental impairment (if present) had been assessed between ages 4–8 years old (19). However, this decision-making aid presents both mortality and disability rate data for survivors within the one infographic using various colour schemes and different sized dots within dots to represent varying levels of neurodevelopmental impairments. Within an emotionally intense, potentially time-limited clinical situation, this infographic may be difficult to interpret and synthesise into the decision-making process. Whilst decision-making aids represent a welcome acknowledgement of the importance of parental input into decision-making for these infants, they can be problematic for parents and professionals to rapidly interpret which population the data presented relates to, how closely this resembles their baby and their context, when the presented data was gathered and, in the case of long term morbidity outcomes, over what time period this assessment relates to.

### 4.3 Use of terminology

All the included guidelines, except one, included discussion of palliative care as a potential management option for these infants at birth. The Japanese guideline did not include reference to palliative, comfort or end of life care, which is in keeping with active, intensive care being the standard approach in Japan for infants born from 22 + 0 weeks onwards. However, given the high risks of mortality or morbidity even with provision of intensive care, it would seem appropriate that a palliative approach be integrated into all management plans for infants born at the limits of viability. Palliative care is not synonymous with end-of-life care. Rather, it is a holistic approach to an infant and their family which ensures there is a focus on providing care which includes active management of pain and distress and integration of the infant and family's physical, emotional, social and spiritual needs. Palliative care can be integrated alongside provision of full and active intensive care, particularly in cases where the prognosis is uncertain. This position, and the importance of professionals understanding that palliative and end-of-life care are related but distinct entities, is being emphasised more emphatically in recent neonatal professional documents, such as the recent BAPM publication, “Recognising Uncertainty: An integrated framework for palliative care in perinatal medicine” (27).

For the other guidelines included in this review, comfort care was presented as an option for management at birth for periviable infants with the wording of comfort care being used interchangeably with palliative care. However, use of the word “palliative” in this context is inaccurate. For infants born at these extremely preterm gestations, the infant will not feasibly be able to survive to discharge without intensive care provision. Therefore, in these circumstances when discussing comfort care what is being described is end-of-life care. This is important not simply for semantic reasons. As stated above, it is important for perinatal professionals to understand and use language which accurately acknowledges the distinctions between palliative and end of life care. They should not be used interchangeably. This adjustment in language is required to ensure perinatal professionals do not associate implementation of palliative care principles and practices as only relevant in an end-of-life situations (28). More pertinently in the context of periviable birth, it is important that there is clarity about what comfort care at birth involves and what the outcome of comfort care will be: the death of the infant in the delivery room (29). Parents should be provided with clear information, compassionately delivered, regarding what the dying process may look like for their periviable baby and the measures that can be taken to ensure their baby is comfortable during this process and the options they have for memory making in their specific context. These elements of care constitute high quality end of life care (30).

Parents should have clarity about when perinatal professionals mean end of life care and when they mean a palliative care approach. This is necessary in order to avoid potential confusion either in regard to management at delivery, or, in cases where the decision has been for intensive care management at birth and the infant is admitted to NICU, parents should not be left to equate the involvement of palliative care practices and/or teams with an implication that this means their baby is actively dying. This clarity can only be achieved with the accurate use of terminology by perinatal professionals.

### 4.4 Strengths and limitations

A strength of this review is the structured approach taken to selecting countries for inclusion. By utilising the OECD listings, the review is reflective of those countries with the highest spend on healthcare (% GDP) therefore, differences in approaches to these infants is unlikely to be predominantly dictated by economic limitations. This allows for exploration of differing sociocultural and moral perspectives and considerations, rather than exclusively economic. Additionally, a structured data extraction matrix was utilised to ensure comparison was possible across the guidelines. This review was also strengthened by inclusion of guidelines written in languages other then English. Utilising online generated translation programs – in this case Google Translate – allowed the authors to interact with guidelines written from all over Europe without incurring expensive translation costs. This has allowed this review to be more inclusive and comprehensive.

The review was limited by the variation in presentation of the different national guidelines. For some countries, there was no unifying professional guideline or framework (France and Belgium), and in these cases consensus statements or professional guidelines relating to the largest geographical area was used. For example, for Belgium, reflective of the fact that it is composed of three autonomous regions (Flanders, Wallonia and Brussels) there is not one national guideline for periviable birth management (31). Instead, the Flanders region has a professional guideline, whereas the Wallonia and Brussels regions have local, institutional guidance (20). Therefore, for the purpose of this review, the Flanders guideline was selected for inclusion as this represented a regional approach, rather than a local approach. Due to this variation, there were differing formats of the included guidelines. The structured data extraction matrix aided consistency of information collated from the included guidelines, as did use of the AGREE II tool for the critical appraisal of quality.

## 5 Conclusions

There is marked variation in recommended practice in relation to the definition of and management for periviable infants across the countries included in this review. This is despite all the included countries having the highest spend on healthcare as a proportion of their GDP. Whilst this statement alone does not necessarily translate into highest spend on perinatal services, it is an indicator that the variation in recommendations seen in the review is likely not reflective of limited means to provide intensive care to these infants, but rather reflects the differing sociocultural contexts. Periviable birth inherently requires a balance of provision of intensive care in the hope of survival against the risk that this intensive care is overtly detrimental, either in terms of mortality or suffering and significant, lifelong morbidity risks. Where the balance of these considerations lies will be different for each individual and may be influenced by, and reflective of, the moral culture and priorities of different societies. Given the significant variation in outcomes for these infants and the substantial impact and implications of periviable birth on their parents and wider families, it would seem appropriate that the value system of that individual family is included within the decision-making process for the individual infant. This is reflected in the shared decision-making approach encouraged by nine of the ten reviewed guidelines.

Although some of the guidelines did provide decision-making aids for parents and perinatal professionals, most guidelines contained scant information about how best to convey and discuss the spectrum of potential outcomes, risks, uncertainties and hope that are intrinsic to the periviable decision-making process. More research is needed to delineate how to optimally approach these conversations to meaningfully involve parents in decisions about their baby's care and determine the most relevant outcome measures that parents with lived experiences of periviable birth would deem most important.
